# Placental Adaptive Changes to Protect Function and Decrease Oxidative Damage in Metabolically Healthy Maternal Obesity

**DOI:** 10.3390/antiox9090794

**Published:** 2020-08-26

**Authors:** Celeste Santos-Rosendo, Fernando Bugatto, Alvaro González-Domínguez, Alfonso M. Lechuga-Sancho, Rosa Maria Mateos, Francisco Visiedo

**Affiliations:** 1 Department of Biology, University of Cádiz, 11519 Cádiz, Spain; celeste.santos@uca.es; 2 Inflammation, Nutrition, Metabolism and Oxidative Stress Study Group (INMOX), Biomedical Research and Innovation Institute of Cádiz (INiBICA), Research Unit, University Hospital “Puerta del Mar”, 11009 Cádiz, Spain; fernando.bugatto@uca.es (F.B.); alvaro.gonzalez@inibica.es (A.G.-D.); alfonso.lechuga@uca.es (A.M.L.-S.); 3 Area of Pediatrics, Department of Child and Mother Health and Radiology, Medical School, University of Cádiz, 11003 Cádiz, Spain; 4 Division of Fetal-Maternal Medicine, Department of Obstetrics and Gynecology, Puerta del Mar University Hospital, 11009 Cádiz, Spain; 5 Pediatric Endocrinology Unit, Department of Pediatrics, Puerta del Mar University Hospital, 11500 Cadiz, Spain; 6 Area of Biochemistry and Molecular Biology, Department of Biomedicine, Biotechnology and Public Health. University of Cádiz, 11519 Cádiz, Spain

**Keywords:** oxidative stress, nitrosative stress, placenta, obesity, antioxidant, nitration, pregnancy-related disorder

## Abstract

Pregnancy-related disorders, including preeclampsia and gestational diabetes, are characterized by the presence of an adverse intrauterine milieu that may ultimately result in oxidative and nitrosative stress. This scenario may trigger uncontrolled production of reactive oxygen species (ROS) such as superoxide anion (O^●−^) and reactive nitrogen species (RNS) such as nitric oxide (NO), along with an inactivation of antioxidant systems, which are associated with the occurrence of relevant changes in placental function through recognized redox post-translational modifications in key proteins. The general objective of this study was to assess the impact of a maternal obesogenic enviroment on the regulation of the placental nitroso-redox balance at the end of pregnancy. We measured oxidative damage markers—thiobarbituric acid-reacting substances (TBARS) and carbonyl groups (C=O) levels; nitrosative stress markers—inducible nitric oxide synthase, nitrosothiol groups, and nitrotyrosine residues levels; and the antioxidant biomarkers—catalase and superoxide dismutase (SOD) activity and expression, and total antioxidant capacity (TAC), in full-term placental villous from both pre-pregnancy normal weight and obese women, and with absence of metabolic complications throughout gestation. The results showed a decrease in C=O and TBARS levels in obese pregnancies. Although total SOD and catalase concentrations were shown to be increased, both activities were significantly downregulated in obese pregnancies, along with total antioxidant capacity. Inducible nitric oxide sintase levels were increased in the obese group compared to the lean group, accompanied by an increase in nitrotyrosine residues levels and lower levels of nitrosothiol groups in proteins such as ERK1/2. These findings reveal a reduction in oxidative damage, accompanied by a decline in antioxidant response, and an increase via NO-mediated nitrative stress in placental tissue from metabolically healthy pregnancies with obesity. All this plausibly points to a placental adaptation of the affected antioxidant response towards a NO-induced alternative pathway, through changes in the ROS/RNS balance, in order to reduce oxidative damage and preserve placental function in pregnancy.

## 1. Introduction

Obesity is considered a chronic low-grade inflammation with multifactorial etiology and has become a serious public health problem because of its increasing prevalence in low-income countries, especially among middle-aged women [1]. The incidence of obesity is of particular concern among women at reproductive age because of its impact during pregnancy. Maternal obesity is closely related to some obstetric complications and poor pregnancy outcomes, including preeclampsia (PE), gestational diabetes mellitus (GDM), preterm birth, or cesarean delivery, which increase an overall risk of severe morbidity or mortality in the maternal–fetal unit [2,3,4,5,6]. In addition, excessive adiposity or weight gain in pregnancy may have a significantly impact over the intrauterine environment and, consequently, on fetal development and the child’s health later in life by fetal metabolic programming that could ultimately promote an increased risk of developing metabolic syndrome, diabetes, or cardiovascular disease [7,8,9].

Obesity in pregnancy is characterized by the presence of hyperlipidemia and heightened inflammatory state, including higher levels of pro-inflammatory mediators [10,11]. This adverse environment can eventually lead to changes in placental function and, subsequently, complications in fetal development [12,13,14]. The placenta *per se* is an important source of production of pro-oxidant agents in normal pregnancy, mainly due to the presence of a high mitochondrial metabolic activity and the action of enzymes such as NADPH oxidase or xanthine oxidase, responsible for the production of reactive oxygen species (ROS) such as superoxide anion (O_2_^●−^) or hydrogen peroxide (H_2_O_2_). Under normal physiological conditions, these reactive species play important roles as second messengers in the cell signaling regulation. In pregnancy, these pathways can respond to a variety of stimuli or insult related to perturbations in the maternal blood supply to the placenta and inflammation.

On the other hand, the placenta has a complex system of antioxidant response, including superoxide dismutase (SOD) or catalase (CAT) enzymes, which usually maintains the action of ROS in balance [15,16]. When the production of ROS exceeds the endogenous antioxidant defenses, a process known as oxidative stress (OS) occurs. OS is now recognized to play an essential role in certain placental-related disorders in pregnancy such as GDM, PE, and intrauterine growth retardation [17,18,19,20]. Nevertheless, the impact of an obesogenic milieu on placental redox status is still not completely understood. In this line, the maternal obesity associated with metabolic alterations seems to lead to the appearance of an elevated placental OS compromising both placental metabolism and antioxidant status. In contrast, no clear evidence has been found of increased OS in human placenta associated with increasing maternal body mass index (BMI), especially in overweight pregnant women [21,22]. Interestingly, pregravid maternal obesity contributes to the development of placental oxidative stress already in the first trimester of pregnancy [23].

In addition to ROS, the placenta can also generate reactive nitrogen species (RNS) in response to different inflammatory and metabolic mediators. Among them, nitric oxide (NO) is a pleiotropic signaling agent able to regulate key biological processes at the cellular level. An overproduction of NO by the nitric oxide synthase (NOS) enzyme has been widely implicated in the pathogenesis of different disorders, including cancer, diabetes, autoimmune, and cardiovascular diseases, along with pregnancy-related pathologies [24]. In these events, NO disrupts protein signaling pathways by mechanisms termed post-translational modifications (PTMs), including protein S-Nitrosylation and Nitration, which may affect its biological functions by forming NO-derived complexes known as S-nitrosothiol (−SNO) and nitrotyrosine (−NO_2_), respectively. Our group has recently pointed out S-Nitrosylation as a crucial mechanism through which NO specifically regulates certain key placental proteins in pregnancies with GDM [25]. This phenomenon has also been proposed to play a role in the placental pathophysiology in PE, both leading to nitrosative stress [26]. Moreover, the combination of NO and O_2_^●−^ produces peroxynitrites (ONOO^●−^), a powerful pro-oxidant that can exert different deleterious effects by protein nitration, forming nitrotyrosine residues [27]. In this line, many relevants cellular proteins are potential ONOO^●−^ targets, including transporters, enzymes, structural proteins, and signal transduction molecules, affecting protein formation and eventually placental function, and fetal growth and development. This process has been previously identified in the placenta from PE and GDM pregnancies in association with an altered placental function [28,29].

All this seems to indicate that both ROS and RNS are highly present in human placenta under pathological conditions related to pregnancy, causing harmful effects on cellular proteins and leading to loss of function. Unlike other pathological states associated with pregnancy, little attention has been dedicated to the role of placental oxidative and nitrosative stress in cases of pregnancies with maternal obesity, particularly in those with a lack of metabolic complications during the course of pregnancy. In this sense, the overall goal of this study was to evaluate the effects of a metabolically healthy pregnancy with pregravid obesity in the balance of ROS/RNS/antioxidants system in full-term placentas.

## 2. Material and Methods

This study was previously approved by the Ethics Committee of Hospital Universitario “Puerta del Mar” (Cádiz, Spain) with study identification code 04.2012, in accordance with the protocols established in the Declaration of Helsinki. All participants in this study were previously informed and gave their written consent in order to be included as study subjects.

### 2.1. Products and Antibodies

All the chemical products used in this work were purchased from Sigma (St. Louis, MO, USA), Biorad (Hercules, CA, USA), and Thermo Scientific (Madrid, Spain). Primary (anti-ERK1/2, anti-SOD 1, anti-catalase, and β-actin) and secondary antibodies came from Cell Signaling Technology (Danvers, MA, USA). iNOS antibody was from R&D Systems (Minneapolis, MN, USA) and nitrotyrosine antibody was from Merck Millipore (Darmstadt, Germany).

### 2.2. Study Subjects

The study groups consisted of nine normal weight (Lean; pre-pregnancy BMI < 25 kg/m^2^) and eight obese (OB; pre-pregnancy BMI ≥ 30 kg/m^2^) pregnant women who gave birth at the Obstetric and Gynecology unit of University Hospital “Puerta del Mar” (HUPM). All patients included in the study had no history of PE, hypertension disorders, chronic diseases, fetal anomalies, smoking habits, infections, or metabolic complications. Furthermore, the participants were classified with metabolically healthy phenotype (uncomplicated pregnancy) mainly following three criteria proposed by Rey-López et al.: high-density lipoprotein cholesterol, triglycerides, and plasma glucose [30]. BMI ≥ 30 kg/m^2^ was the main indicator used to define maternal obesity. All participants showed a normal glucose tolerance at 24–28 weeks following the criteria and protocols established by the National Diabetes Data Group [31]. The indications for elective caesarean section at term were breech presentation and previous caesarean section.

### 2.3. Tissue Collection and Placental Samples

All placentas included in the study were quickly collected after a scheduled cesarean section in the Obstetrics and Gynecology unit of University Hospital “Puerta del Mar”. Placentas were placed on ice and transported to the research laboratory within 10 min of delivery. Then, decidual tissue and large vessels were removed from villous placenta by blunt dissection on aseptic culture conditions. Afterward, small fragments of villous (~100 mg) were isolated from the central area of the placenta, close to the umbilical cord, washed in cold PBS to remove attached blood, and stored at −80 °C until further analysis.

### 2.4. Protein Extract Preparation

Total proteins were extracted from frozen placental samples following homogenization in lysis buffer in combination with protease inhibitors and phenylmethylsufonyl fluoride (PMSF). After 30 min on ice, extracts were sonicated and centrifugated at 18,000× *g* for 5 min at 4 °C. Then, pellets were removed and supernatants (protein extracts) were used for further analysis. Total proteins were quantified by the bicinchoninic acid method (Thermo Scientific, Madrid, Spain).

### 2.5. Measurement of Total Protein Expression by SDS-Western Blotting Assay

Equal concentrations of solubilized proteins (40 μg/sample) were denatured by heat at 100 °C for 5 min in sodium dodecyl sulfate (SDS) sample buffer, resolved by 6–12% SDS-polyacrylamide gel electrophoresis (SDS-PAGE), transferred to polyvinylidenedifluorid (PVDF) membranes and blotted with primary antibodies directed against the different proteins of study (Catalase, SOD, nitrotirosine, and iNOS) at a dilution of 1:500–1:1000, and secondary antibodies used at a dilution of 1:5000. The incubated membranes were visualized by the chemiluminiescence method using WesternBright Sirius Horseradish Peroxidase (HRP) substrate (Advansta Inc., San Jose, CA, USA). Band intensity for each protein was quantified by the Image-J program (Wisconsin, NIH, USA). The membrane was stripped and re-tested with β-actin as a protein loading control for each of the proteins examined.

### 2.6. Determination of Oxidative Status Markers

#### 2.6.1. Lipid Peroxidation (LPO)

Thiobarbituric acid-reacting substances (TBARS) were measured by the spectrophotometric method described by Buege et al. [32]. The values were expressed as nmol of malondialdehyde (MDA)/mg protein in placental tissue.

#### 2.6.2. Carbonyl Groups (C=O)

For the determination of carbonyl groups in placental tissue, the spectrophotometric dinitrophenyl hydrazine method of Levine et al. was followed for every sample, with a blank for each one [33]. Samples containing at least 0.5 mg extract protein were incubated with 0.3% (*v/v*) Triton X-100 and 1% (*w/v*) streptomycin sulphate for 20 min to remove the nucleic acids and were centrifuged at 2000× *g*. Supernatants (200 μL) were mixed with 300 μL of 10 mM 2,4-dinitrophenylhydrazine (DNPH) in 2 M HCl. The blank was incubated in 2 M HCl. After 1 h of incubation at room temperature, proteins were precipitated with 10% (*w/v*) trichloroacetic acid and the pellets were washed three times with 500 μL of ethanol:ethylacetate (1:1). The pellets were finally dissolved in 6 M guanidine hydrochloride in 20 mM potassium phosphate at pH 2.3, and the absorption at 370 nm was measured. Protein recovery was estimated for each sample by measuring at 280 nm. Carbonyl content was calculated using a molar absorption coefficient for aliphatic hydrazones of 22,000 M^−1^ cm^−1^.

### 2.7. Determination of Antioxidant Defense Markers

#### 2.7.1. Native SDS-PAGE SOD Activity

Total SOD activity in the placental homogenates was evaluated by analysis of the isoenzymatic SOD pattern. Sample proteins were separated by native polyacrylamide gel electrophoresis (PAGE) on 10% acrylamide gels, and isoenzymes were visualized in gels by nitro blue tetrazolium (NBT) staining. SOD isozymes were detected in gels as achromatic bands over a blue background, a consequence of the NBT reduction to formazan blue by the O_2_^●−^ radicals generated in the assay [34].

#### 2.7.2. Catalase Activity

Catalase activity was determinated by spectrophotometric analysis of hydrogen peroxide breakdown at 240 nm, as described by the method of Aebi [35].

#### 2.7.3. Total Antioxidant Capacity

Total antioxidant capacity (TAC) assay was measured by the method described by Erel [36]. Results were expressed as μmol of trolox equivalents per mg of proteins in placental tissue.

### 2.8. Nitrosative Stress Markers

#### 2.8.1. S-Nitrosylated Protein Detection by Biotin Switch Assay

S-nitrosylated proteins were detected using the Biotin-Switch assay described by Jaffrey and Snyder with a few minor modifications [37]. Placental samples (~50 mg) were homogenized in lysis buffer (100 mM Hepes-NaOH, 1 mM EDTA, 0.5 mM PMSF, 1% Triton X-100, 0.1% SDS, and 0.1 mM neocuproine, pH 7.2). Equal amounts of proteins in each lane were determined using the BCA protein kit. 1 mg protein was mixed with 400 μL blocking buffer (25% SDS, 10 mM MMTS in standard buffer, containing 100 mM Hepes-NaOH, 1 mM EDTA, and 0.1 mM neocuproine, pH 7.2) and boiled in agitation at 55 °C in the dark for 30 min in order to allow S-methylthiolation of each cysteine thiol using S-methylmethane thiosulfonate (MMTS). After discarding excess MMTS by precipitation with acetone, protein samples were then reduced to thiols with 50 mM sodium ascorbate and biotinylated with 33 μL reducing buffer (4 mM Biotin-HPDP, 1% SDS in standard buffer). After, the biotinylated proteins were drawn out by neutravidin agarose-beads, eluted by SDS sample buffer at 95 °C for 5 min, and detected by inmmunoblotting by specific ERK1/2 antibodies.

#### 2.8.2. Detection and Quantification of Nitrated Proteins and iNOS by SDS-PAGE Blotting

Equal amounts of protein fractions (40 μg/sample) were dissolved with SDS sample buffer, heated at 100 °C for 5 min, resolved on 10% SDS-polyacrylamid gels, transferred onto PVDF membranes, and immunoblotted with primary antibody anti-nitrotyrosine (diluted at 1:2000), and then with secondary antibody (diluted at 1:5000). Signals were detected using WesternBright Sirius HRP system detection (Advansta Inc., San Jose, CA, USA) and then band intensity was measured using Image J software (Wisconsin, NIH, USA).

### 2.9. Data Analysis

Data were statistically analyzed using the SPSS software (SPSS, Chicago, IL, USA). Comparisons were done by using the Mann–Whitney U-test or Student’s t-test. Results are expressed as mean ± standard deviation (SD) and differences were considered significant when *p* < 0.05.

## 3. Results

### 3.1. Clinical and Anthropometric Characteristics

The anthropometric and clinical parameters of the participants evaluated in study groups are shown in Table 1. Both groups did not show significant differences related to all, with the exception of the maternal BMI variables, which reflected a significant increase in obese group.

### 3.2. Assesesment of Placental Antioxidant Defense Markers

**Figure 1:**
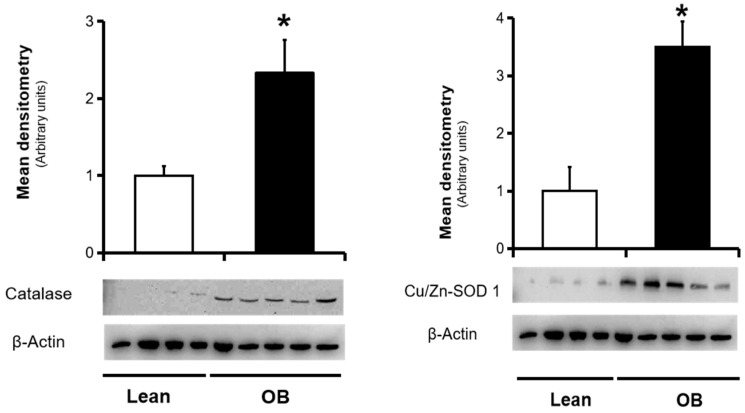
Total concentration of endogenous enzymatic antioxidants (catalase and Cooper/Zinc SOD1) in full-term placentas from normal weight (lean; n = 9) and obese (OB; n = 8) women. These antioxidant markers were assessed in placental protein extracts by Western blot analysis, as described in the “Material and Methods” Section. Top: the *y*-axis represents the ratio of total antioxidant versus β-actin in arbitrary units. Bottom: a representative picture of all Western blots is shown. Results were reported as means ± SD. * p < 0.05 relative to the lean group.

**Figure 2:**
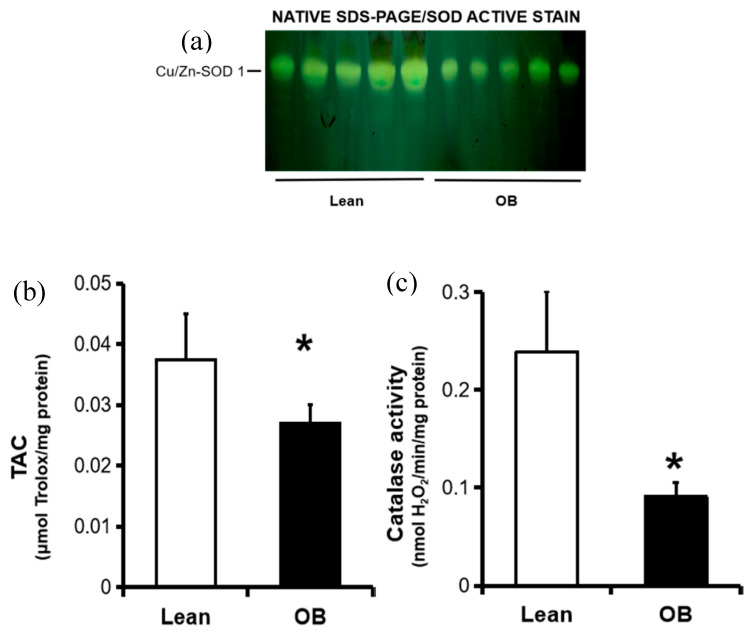
Endogenous antioxidant activity in full-term placentas from normal weight (lean, n = 9) and obese (OB, n = 8) pregnancies. (**a**) SOD activity pattern was obtained by native SDS-PAGE on 10% (w/v) polyacrylamide gels, and then stained by the photochemical nitroblue tetrazolium method. For each sample, 40 μg of protein was loaded per lane. A representative picture of all analyses of the isoenzymatic SOD activity is shown. (**b**) Catalase activity and (**c**) TAC were quantified by spectrophotometric analysis, as described in the “Material and Methods” Section. Results were reported as mean ± SD of enzymatic activity. * *p* < 0.05 relative to the lean group. Cu/Zn SOD: Copper/Zinc Superoxide dismutase; SDS-PAGE: sodium dodecyl sulfate-polyacrylamide gel electrophoresis; TAC: total antioxidant capacity.

Endogenous defense against an abundance of pro-oxidant agents involves an overall action of antioxidant enzymes in order to detoxify free radicals and thus avoid possible tissue damage. Regarding the main antioxidant enzymes, SOD 1 catalyzes the conversion of the O_2_^●−^ radical to H_2_O_2_, and then, cytosolic catalase transforms H_2_O_2_ to water. In placenta, there are two SOD isoforms, copper-Zinc-containing SOD1 (Cu/Zn-SOD) at the villous stroma, and manganese-containing SOD2 (MnSOD) at the villous endothelium, both acting as the first line of antioxidant defense. The expression of Cu/Zn-SOD and catalase is constitutive, although it may be stimulated by pro-oxidants, including NO. In placental homogenates, SOD and catalase protein levels were found to be increased by Western blotting in obese women (Figure 1). Converserely, their activities were found diminished in the obese group, along with total antioxidant capacity (Figure 2).

### 3.3. Assesesment of Placental Oxidative Damage Markers

**Figure 3:**
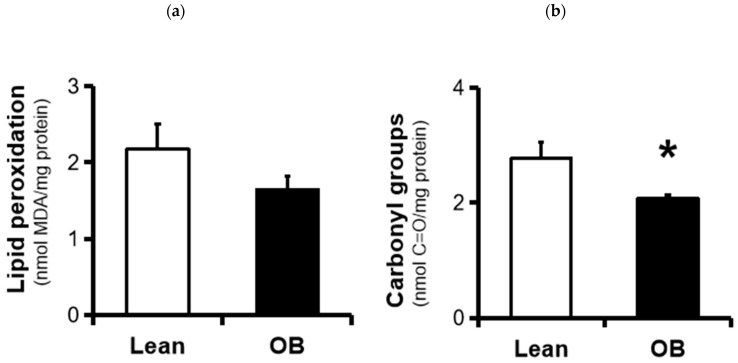
Markers of oxidative damage in full-term placentas from normal weight (lean, n = 9) and obese (OB, n = 8) pregnancies. (**a**) TBARS levels and (**b**) carbonyl groups concentration were assessed in placental protein homogenates using colorimetric techniques, as described in the “Material and Methods” Section. Results were reported as means ± SD of oxidative markers. * *p* < 0.05 relative to the lean group. C=O: carbonyl groups; MDA: malonaldehyde; TBARS: Thiobarbituric acid-reacting substances.

We further investigated two universal parameters related to the generation of key pro-oxidants involved in the development of OS. Pro-oxidant agents attack cell membrane phospholipids and react with polyunsaturated fatty acids to form lipid peroxides, an indicator of lipid oxidation. In obese placentas, a non-significant trend towards decreased TBARS levels as a lipid peroxidation biomarker was found (~25%, *p*-value = 0.10) (Figure 3a). On the other hand, protein carbonyls formation, a hallmark of protein oxidation specially caused by O_2_^●−^, showed a significant decrease in placentas from obese pregnancies (~30%, *p*-value = 0.045).

### 3.4. Nitrosative Stress Markers

**Figure 4:**
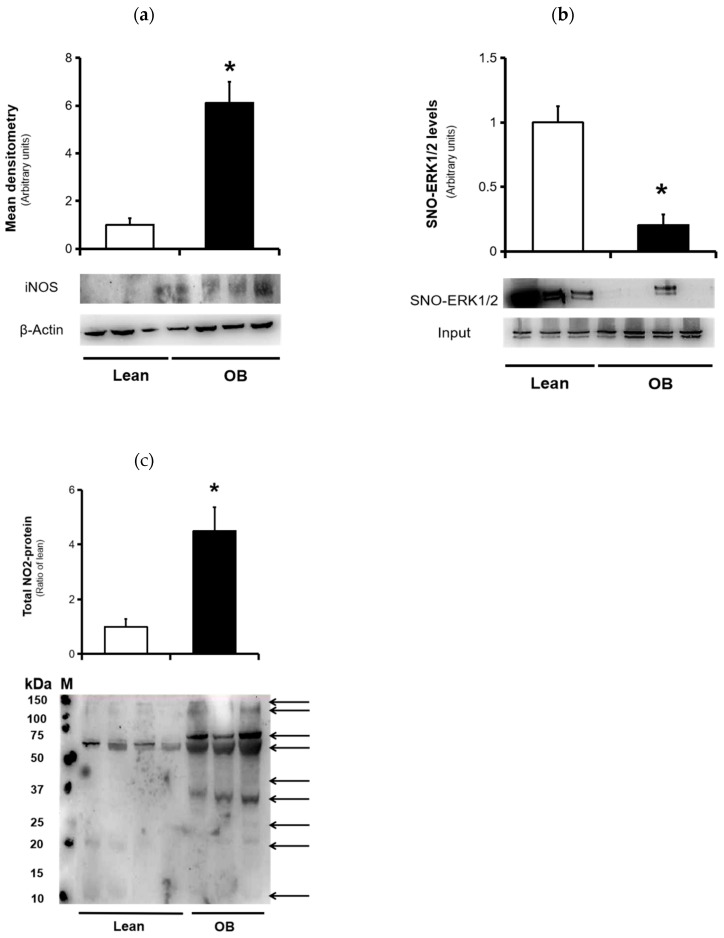
NO-induced nitrosative stress markers in placental samples from normal weight (lean, n = 9) and obese (OB, n = 8) pregnancies. (**a**) iNOS expression levels. This enzyme was assessed in placental protein extracts by Western blot analysis, as described in the “Material and Methods” Section. (**b**) Detection and quantification of ERK nitrosylated (SNO-ERK1,2) levels. The placental extracts were processed and subjected to the Biotin-Switch method. The biotin-labeled SNO proteins were further subjected to neutravidin capture, separated by using 10% by SDS-PAGE, and immunoblotted with an anti-ERK antibody. Total ERK were represented as total protein (input). (**c**) Detection of nitrated proteins by immunoblot analysis using antinitrotyrosine antibodies. In each lane, all nitrated proteins were counted in order to quantify the total nitration protein level for each placental sample. Molecular weight markers (M) and sizes (kDa) are shown, and also possible targets of nitrated proteins (arrows) are indicated. In all cases: top represents the mean densitometry expressed in arbitrary units and bottom shows a representative picture of all Western blots. The results were expressed as means ± SD. * *p* < 0.05 relative to the lean group or input. iNOS: inducible nitric oxide synthase.

iNOS expression, a marker of inflammation and NO production, showed a significant increment in expression levels in human placenta from the obese pregnant compared to lean group (Figure 4a), demonstrating that inducible isoform of NOS is activated in villous tissue under pathological conditions, such as maternal obesity. On the other hand, formation on S-Nitrosothiol groups, termed as S-Nitrosylation, has been widely recognized as a main actor in cellular signaling pathways, especially associated to activation of proteins of cell survival. Thus, the ERK1/2 pathway is highly regulated by action of NO through mechanisms related to PTMs in various pathophysiological conditions. We used the Biotin-Switch assay in order to measure the levels of placental ERK1/2-SNO in both groups, and a significant decrease was found in placental tissue from pregnant women with obesity (Figure 4b). An increased NO production, with reduced S-Nitrosylation, suggests alternative NO-mediated regulation mechanisms, such as protein nitration. To assess this, we determined the nitration protein profile in full-term placentas of both normoweight and obese pregnancies. Equal amounts of placental protein extracts were detected via immunoblotting with anti-nitrotyrosine antibody. As shown in Figure 4c, total NO_2_-proteins levels increased significantly in obese placentas compared to those in the lean group.

## 4. Discussion

The incidence of obesity before pregnancy is quickly increasing worldwide in the 21st Century and is currently considered a chronic inflammatory condition that may negatively affect both mother and fetus, and their offspring [38,39]. The placenta appears to play an essential role during gestation given its intermediate situation and regulatory functions. Thus, this organ may respond to perturbations in the intrauterine environment throughout pregnancy, even at early stages of pregnancy, with negative consequences for the development of the fetus [40]. In this sense, an obesogenic millieu can lead to overproduction of ROS and RNS, which may ultimately lead to changes in the normal structure and function of the placenta. Therefore, a right balance between pro-oxidants agents (H_2_O_2_, O_2_^●−^, or NO), antioxidant systems (catalase, SOD), as well as pro-oxidative enzymes (NADPH and xanthine oxidase or nitric oxide synthases) seems crucial in maintaining placental homeostatic capacity to initiate a normal adaptive response during pregnancy [41]. Our findings highlight the existence of an imbalance of the antioxidant/ROS/RNS system in human placenta from pregnancies with pregravid maternal obesity without associated metabolic complications during gestation, which is characterized by a failure of the antioxidant response, along with a decrease in oxidative damage and an enhanced NO-induced nitrosative stress, predominantly by abundance of nitrotyrosine groups via protein nitration.

At the beginning of pregnancy with pregravid obesity, a chronic inflammation may activate a set of mechanisms that ultimately form an adverse intrauterine inflammatory environment throughout pregnancy. In this sense, the accumulation of a heterogeneous population of macrophages and pro-inflammatory mediators have been found in placenta from obese pregnancies [42]. On the other hand, placental mitochondrial dysfunction has been observed with increased maternal adiposity, along with exacerbated ROS production [17]. The main physiological source of ROS in placenta is the NADPH oxidase (NOX) family, especially NOX1 and NOX5 isoforms, identified in syncytiotrophoblast and vascular endothelium [43]. Other enzyme systems such as xanthine oxidase and the mitochondrial respiratory chain are also involved. Hernandez et al. demonstrated that both NOX isoforms are the most relevant source of placental superoxide anion, and also, the NOX activity is higher in the first trimester, along with increased antioxidant activities (e.g., SOD, catalase, or glutathione peroxidase) [44]. Moreover, an adipogenic and inflammatory state found in certain physiological pregnancies have been related to changes both in the pro-oxidant and antioxidant defense levels [45,46].

Although OS has been observed in the normal development of the placenta, it clearly seems to be involved in the pathophysiology of pregnancy-associated complications such as PE and GDM [19,20,47,48]. However, studies on OS in maternal obesity are scarce and controversial [17,21,22]. In this sense, our approach has been the assessement of placentas from obese women without any medical complications or metabolic factors, including GDM or insulin resistance. Furthermore, all pregnant women were eligible regarding mode of delivery (elective Caesarean section) in order to avoid the potential impact of labor on placental OS. Also highlighted is the fact the all patients were elegible as metabolically healthy pregnancies based on three biochemical parameters such as plasma glucose, cholestherol and tryglicerides levels, which exhibited normal physiological levels in pregnancy.

In the present study, we first observed a significant decrease in the activity of catalase and SOD antioxidant enzymes in full-term placenta, in spite of an increase in its concentrations, as well as a reduction in total antioxidant capacity (Figure 1 and Figure 2). In parallel, our results show a strong trend of decreased oxidative damage characterized by a decrease in the levels of universal OS markers such as TBARS and carbonyl groups (Figure 3). This overall data reveals the existence of a placental inactivation of antioxidant system response, which would eventually lead to elevated OS in obese women’s pregnancies. However, this conflicting condition is not accompained by a subsequent increase of oxidative damage, including those involved in lipid peroxidation and protein oxidation. This was partially in agreement with other authors who showed lower carbonyl groups formation in placenta of pregnant women with overweight, but not in obese pregnant women, in addition to an unaltered SOD activity among groups [22]. In contrast, Malti et al. found OS-induced oxidative damage in placenta of obese mothers, including showing a high antioxidant activity [21]. In relation to other pregnancy-associated disorders, a reduced enzymatic capacity and increased oxidation has been demonstrated in placenta tissue from preeclamptic women, contributing to the pathogenesis of this gestational disorder [49]. On the other hand, increased oxidative stress in late gestation was observed in several pregnancy-related disorders in association with increased trophoblast apoptosis and altered placental vascular endothelium [50]. Meanwhile, other promising studies have revealed the presence of possible alterations in ROS/SOD ratio based on upregulation of the SOD along with the activation of the superoxide anion degradation pathway at the vascular level in cardiovascular diseases [51].

NO is an essential pleiotropic agent that, under normal physiological conditions, is involved in the regulation of different biological processes, including cell respiration, apoptosis, and proliferation of cells, vascular tone, or gene expression. In human reproductive physiology, NO plays an important role in expression-dependent events of certain signaling molecules (e.g., vascular endothelial growth factor-VEGF), including angiogenesis or vasculogenesis [52]. Thus, an appropriate placental vascularization is necessary to ensure a correct fetal development throughout the gestation. During gestation, NO is synthesized by various NOS isoforms, including endothelial (eNOS) and inducible (iNOS) NO synthases. iNOS expression has been clearly demonstrated in human placenta, so it might play an important role in placental pathophysiology [53]. In this sense, an excessive NO production by the inducible iNOS enzyme has been widely implicated in the pathogenesis of various neurodegenerative, inflammatory, or metabolic diseases [24,54]. Thus, some authors suggest that maternal diabetes is related to an increased expression of the iNOS enzyme and consequently, NO-induced nitrosative stress and cell death [55,56]. Meanwhile, an increase in the production of NO has been reported in pregnancy in which the placenta is the main target of these perturbations [57]. Thus, S-Nitrosylation is a well-known post-translational regulation mechanism by which NO can directly modulate protein function, resulting in altered protein activity and adverse biological consequences [58]. We have previously found an increase of NO-related nitrosative stress by detection of SNO proteins involved in key cellular physiological events, such as cell death, the antioxidant system, and cell signalling [25]. In the present study, we found increased iNOS expression in full-term placental tissue from the obese mothers (Figure 4a), which is in line with other reports [59,60]. On the other hand, cell signaling-related proteins have been described as potential targets of S-nitrosylation, including ERK1/2 [24], which may be essential in regulation of placental processes such as cell replication and trophoblast differentiation. In addition, several studies have highlighted a major role of S-nitrosylation ERK1/2 in various pathophysiological models, which indicates that protein S-Nitrosylation could be crucial for progression of knowledge of NO biology in pregnancy [61]. In this sense, our data show a significant decrease in nitrosothiol group levels in ERK1/2 protein (Figure 4b), thereby reducing S-Nitrosylation in placentas from obese mothers and, consequently, NO bioavailabilty in other relevant processes. In addition, we measured the concentration of phosphorylated ERK1/2 (active form), showing a notably increased activation (data not shown), which seems to indicate that a compensatory regulatory mechanism may be occurring oriented towards the activation of processes related to cell survival rather than cell death, possibly in order to protect the cell function.

Protein nitration, another NO-induced PTM, is extensively present in certain pathological states and systems [27,62,63]. An excess of free radicals can result in the generation of ONOO^-^, a reactive intermediate of the interaction between the NO and O_2_^-^ anion. This potent pro-oxidant agent has a relatively high half-life and and it is able to induce a damage in membrane lipids or modify proteins by PTMs, particularly at the tyrosine residues, leading to the formation of nitrotyrosine groups [24]. Formation of these nitrotyrosine residues has been widely documented as a hallmark of nitrative stress [64]. During gestation, nitrotyrosine generation in placenta has been identified in complicated pregnancies, especially PE and GDM, and recognized as primarily responsible for the increase in maternal and perinatal mortality [28,65]. In this sense, our results show the existence of higher levels of protein nitration in placenta tissue of uncomplicated obese mothers, possibly by modification of placental proteins linked to several biological pathways (Figure 4c). An altered placental nitro-proteome, characterized by the accumulation of nitrotyrosine residues in key proteins, could strongly participate in the placental pathophysiology in pregnancy-related disorders, including maternal obesity [66,67]. Other previous works support this hypothesis of nitrotyrosine action in protein and placental villous (especially in syncytiothrophoblast), or that vascular endothelium may ultimately alter proteins and placental functions [28,29,68,69,70] and contribute to the occurence of long-term metabolic and cardiovascular diseases, both in the mother and in offspring.

## 5. Conclusions

**Figure 5:**
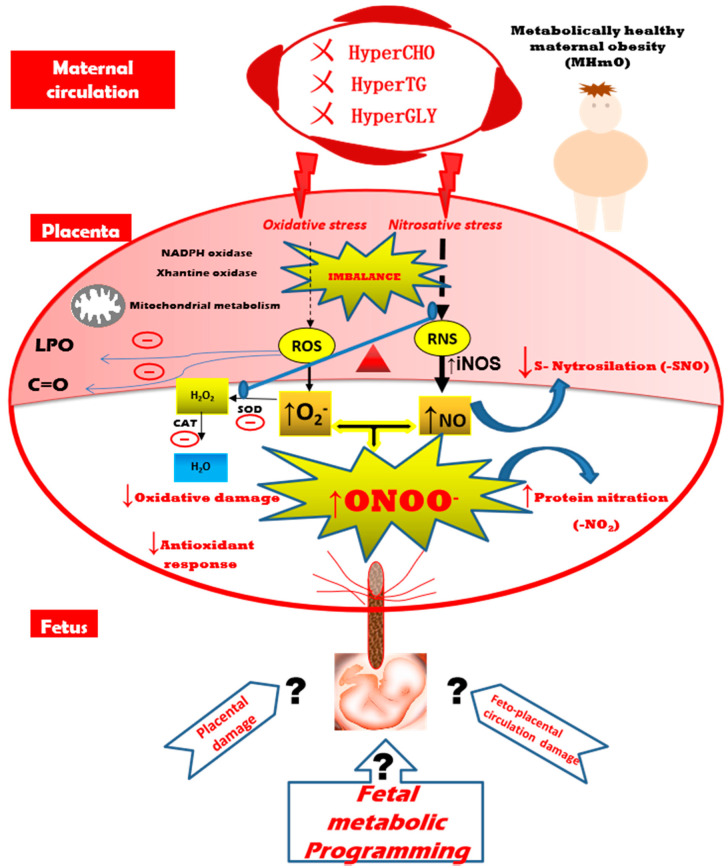
Proposed model of formation of a placental antioxidant/ROS/RNS unit imbalance in pregnancy with metabollicaly healthy maternal obesity. ROS: reactive oxygen species; RNS: reactive nitrogen species; TG: Triglycerides; NO: nitric oxide; iNOS: inducible nitric oxide synthase; SOD: superoxide dismutase; CAT: catalase; O_2_^●−^: superoxide anion; H_2_O_2_: hydrogen peroxide; ONOO^●−^: peroxynitrite; LPO: lipoperoxides; C=O: carbonyl groups; HyperCHO: hypercholesterolemia; HyperTG: hypertriglyceridemia; HyperGLY: hyperglycemia; **㐅** symbol: absence of.

A redox imbalance occurs when the antioxidant response is overflowed, consequently leading to the activation of alternative pathways that, under normal conditions, remain inactive. In this context, we first suggest that the protein nitration may be playing a positive regulatory role, which, if exceeded, could lead to damage in human placenta. Moreover, we secondly propose that an adaptive mechanism of the placenta could be occurring in a healthy maternal obesogenic context. Thus, a failure in the balance of antioxidant/ROR/RNS unit could result in an increased NO-induced nitrosative stress, accompanied by an inactivation of antioxidant systems and a reduction of oxidative damage, thus redirecting the superoxide radicals accumulated along with NO to the formation of peroxynitrite, and ultimately to an increase in protein nitration (Figure 5). Therefore, it seems that NO-induced nitrosative stress preceeds oxidative stress in pregnancies from metabolically healthy maternal obesity. Further studies are needed to reveal the possible targets of NO and peroxynitrite action on key proteins in pregnancy-related disorders such as maternal obesity, in order to analyze the possible effects in the placenta function and, consequently, in fetal development and programming.

## Figures and Tables

**Table 1:** Clinical and anthropometric characteristics in normal weight and obese pregnant women.

Characteristics	Lean Group (n = 9)	Obese Group (n = 8)
Delivery mode	Caesarean section (No labor)	Caesarean section (No labor)
Maternal age (years)	33.1 ± 6.3	35.5 ± 6.4
Gestational age at delivery (weeks)	38.3 ± 1.0	38.3 ± 0.8
Pregravid Maternal BMI (kg/m^2^)	22.5 ± 1.3	35.5 ± 6.4 *
Maternal BMI at delivery (kg/m^2^)	28.1 ± 2.5	37.3 ± 5.7 *
Birthweight (g)	3182 ± 214	3170 ± 336
F/M sex ratio	5/4	5/3
Placental weight (g)	603 ± 75	604 ± 54

Data are given as mean ± standard deviation (SD). * *p*-value < 0.05 when compared to lean group by Mann–Whitney U-test. BMI: body mass index; F/M: females/males.
